# Primary cerebral malignant melanoma in insular region with extracranial metastasis: case report and review literature

**DOI:** 10.1186/s12957-016-0965-7

**Published:** 2016-09-01

**Authors:** Marta Troya-Castilla, Santiago Rocha-Romero, Yamin Chocrón-González, Francisco Javier Márquez-Rivas

**Affiliations:** Neurosurgery Department, University Hospital Virgen del Rocío, Av Manuel Siurot s/n, 410013 Seville, Spain

**Keywords:** Primary, Cerebral, Melanoma, Bleeding, Metastasis

## Abstract

**Background:**

Primary brain melanomas are very infrequent and metastasis outside central nervous system very uncommon. There are some cases in the literature about primary melanoma in the temporal lobe; nevertheless, the insular location has never been described.

**Case presentation:**

The patient presented as left insular intraparenchymal hematoma with multiple bleedings. Complementary tests did not show any tumoral nor vascular pattern in relation with these bleedings. A complete surgical resection was performed, and the diagnosis of malignant melanoma, with BRAF mutation, was obtained after histology exam. Extension studies were negative for skin or mucous melanoma. 18F-FDG PET/CT was performed and a metastatic lymph node was found. The diagnosis was primary brain melanoma with extracerebral metastasis. Dabrafenib 150 mg/12 h was the only chemotherapy during 5 months. After that, Trametinib 2 mg/24 h was added to the treatment. Eighteen months after surgery, the patient is independent, with stable situation, and without new metastasis.

**Conclusions:**

Although malignant melanomas have poor prognosis, total surgical resection and new therapies are increasing the overall survival and improving quality of life. In a patient with suspected brain melanoma, in spite of having extracerebral metastasis, aggressive treatment may be considered.

## Background

Primary cerebral melanomas are extremely infrequent. Only 1 % of primary melanomas come from the brain [1, 2]. Melanoblasts, precursors of melanocytes, are of neural crest origin. During development they migrate to the skin, uvea, mucose membrane, and leptomeninges of the central nervous system (CNS) [3]. Primary brain melanoma arise once the melanocytes of the leptomeninges become neoplastic cells.

The most common locations of primary CNS melanoma are the anterolateral face of the spinal cord and posterolateral face of the trunk. Total excision of brain melanoma is the mainstay of the treatment in most cases [4–6]. Skin melanomas have an aggressive behavior and tend to metastasize to other organs [7]; nevertheless, extracerebral metastasis from primary brain melanoma is extremely rare [8].

We present a patient with primary CNS focal melanoma in a rare location with an inguinal lymph node metastasis and good prognosis after 18 months of diagnosis.

## Case presentation

**Fig. 1:**
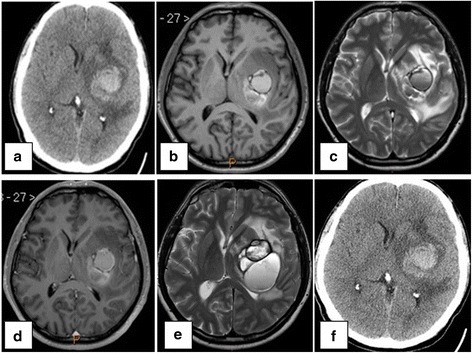
**a** Initial CT scan with left insular hematoma. **b**–**d** MRI-T1, T2, and T1 contrast, respectively, with multiloculate left insular hematoma, heterogeneous signal, and edema. **e** Follow-up MRI-T2 with new bleeding and higher mass effect. **f** Urgency CT scan with higher edema and mass effect

A 41-year-old man came to the hospital with right paresis and minor dysphasia. A CT scan showed a left temporoparietal lesion with intra-extra bleeding, edema, and half-line shifting (Fig. 1a). The MRI displayed a 40-cc multilocular hemorrhage located in the left insular region with bleedings in several stages. The lesion was heterogeneous in every sequence (T1, T2, and T1 with enhancement), with edema and large mass effect without evidence of an underlying lesion (Fig. 1b–d). The cerebral angiography did not show vascular malformations nor tumoral pattern. No peripherally lesion was found in the extension CT scan and physical exam. The neurological deficit improved, and a conservative management was decided until the diagnosed were more certain.

**Fig. 2:**
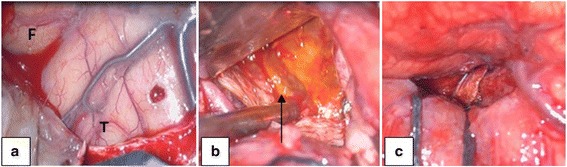
**a** Frontal lobe (F) and temporal lobe (T). The beginning of approach in the Sylvian fissure. **b** After insular corticotomy, the hemorrhagic lesion was found. **c** Complete resection of the lesion

Control MRIs showed new re-bleedings, higher volume and more half-line shifting (54.89 and 75.55 cc of insular hematoma in two controls MRI) (Fig. 1e). During the follow-up, the patient suffered deterioration of his paresis and dysphasia. The CT scan revealed an increase in the mass effect of the lesion (Fig. 1f). Due to an obvious clinical and radiological worsening, a complete surgical resection of the lesion was made (Fig. 2).

**Fig. 3:**
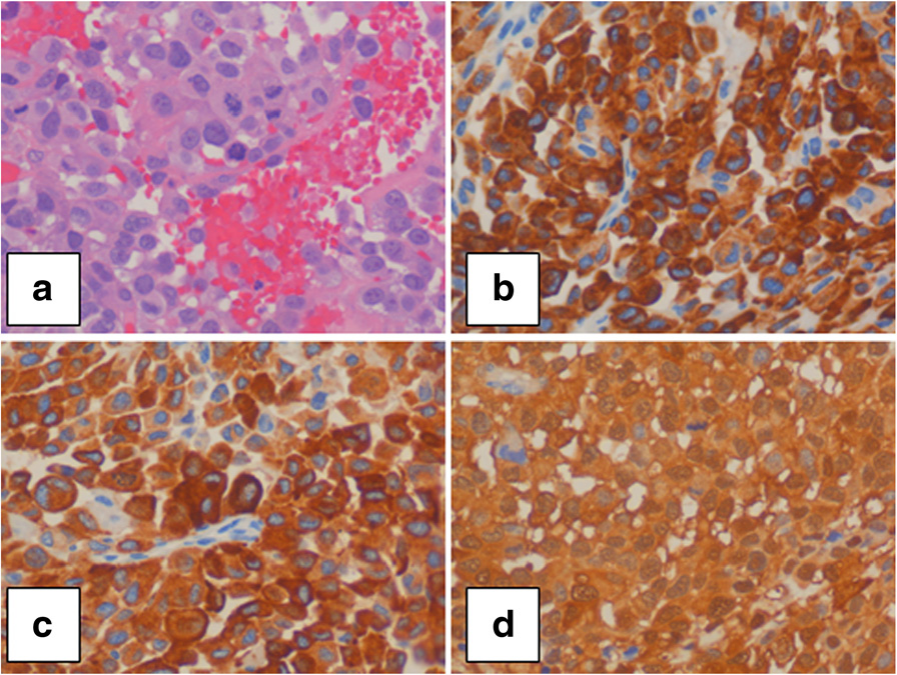
**a** HE ×40. **b** HMB45 ×40. **c** MELAN A ×40. **d** S100 ×40

After surgery, the patient improved neurologically and suffered no complications. The histological study showed up a malignant melanoma with mutation of exon 15 in the BRAF gen (Fig. 3). No primary lesions were found in the skin and mucosa study nor in the ophthalmological exploration. Since there were no primary lesions nether other CNS lesion, the patient was diagnosed of primary cerebral melanoma and was transferred to oncology to complete extension study and adjuvant therapy.

**Fig. 4:**
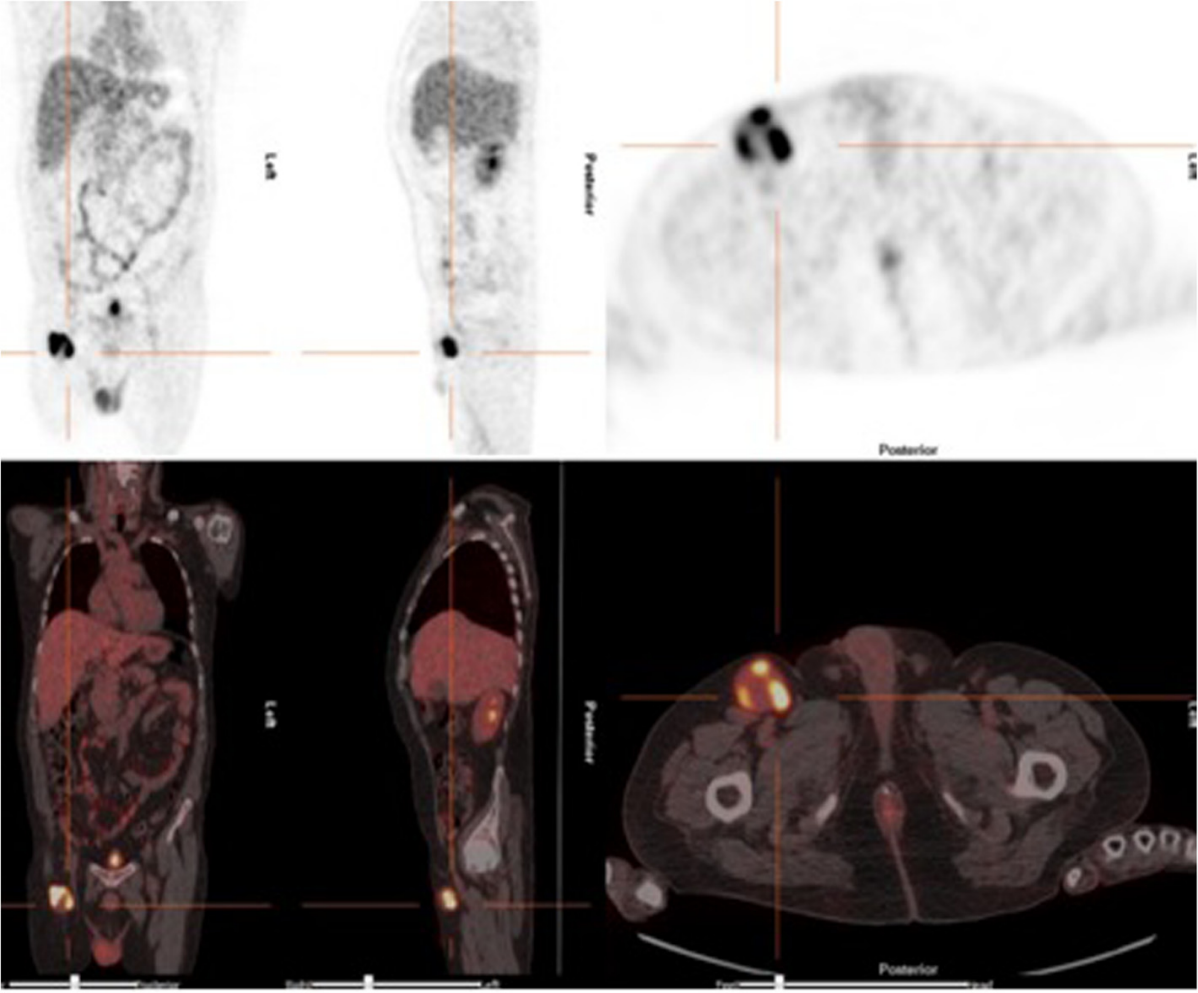
The 18F-FDG PET/CT revealed a pathological deposit in one inguinal lymph node at the right side. The dimension of the lymph node was 4.2 cm × 3.8 cm × 3.5 cm. No other metastases

As FDG PET has shown to be sensitive for assessing metastases in lymph node and for detecting occult distant metastases in patient with malignant melanoma, 18F-FDG PET/CT was performed during the follow-up [9]. The 18F-FDG PET/CT revealed a pathological deposit in one inguinal lymph node at the right side. The dimension of the lymph node was 4.2 cm × 3.8 cm × 3.5 cm in the 18F-FDG PET/CT (Fig. 4).

**Fig. 5:**
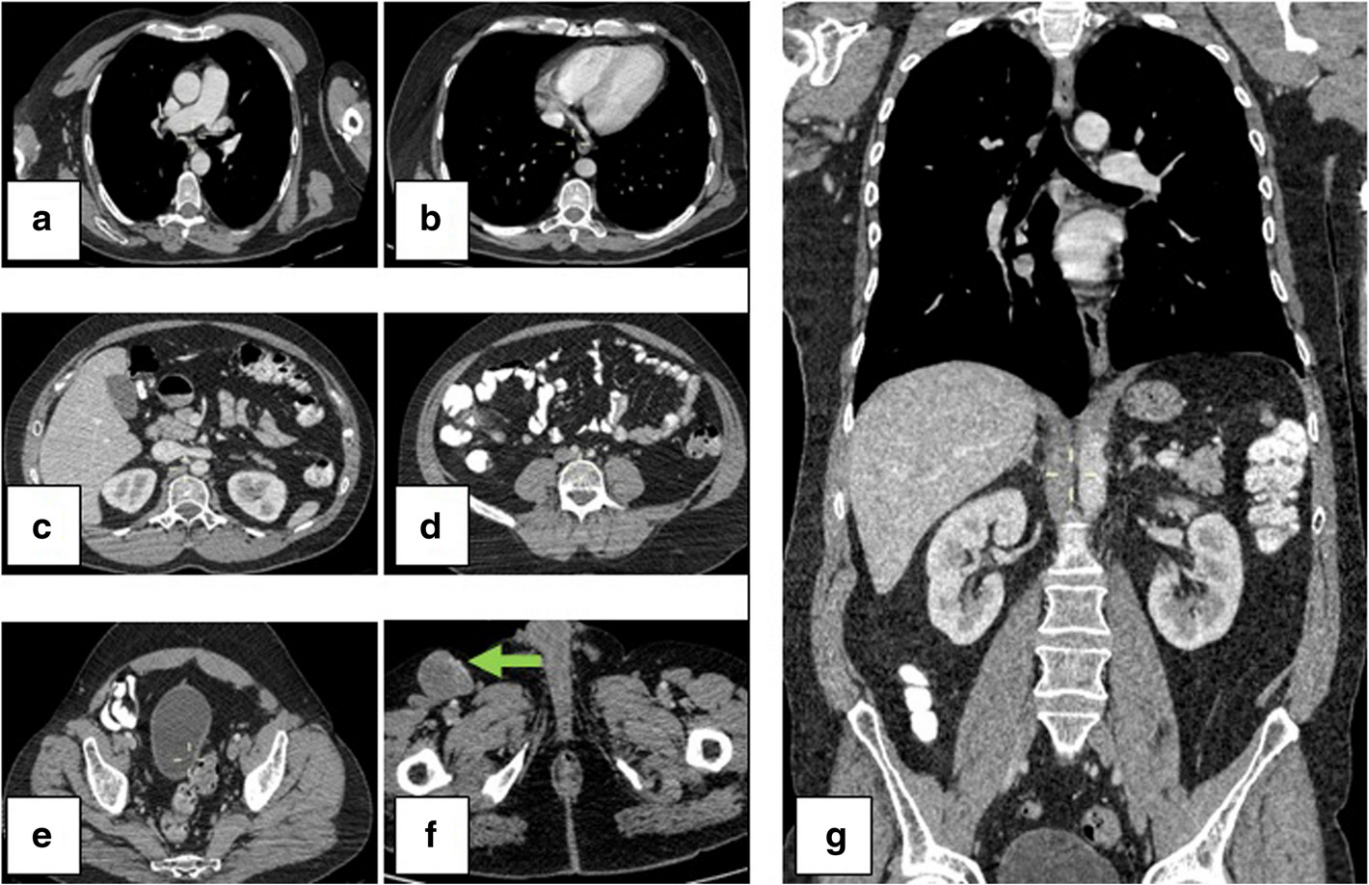
Control CT scan after 17 months of surgery. **a**–**e** Axial thoracic and abdominal section without new lesions. **f** Metastatic right inguinal lymph node, 32 × 22 cm. **g** Coronal thoracic and abdominal section without lesion

**Fig. 6:**
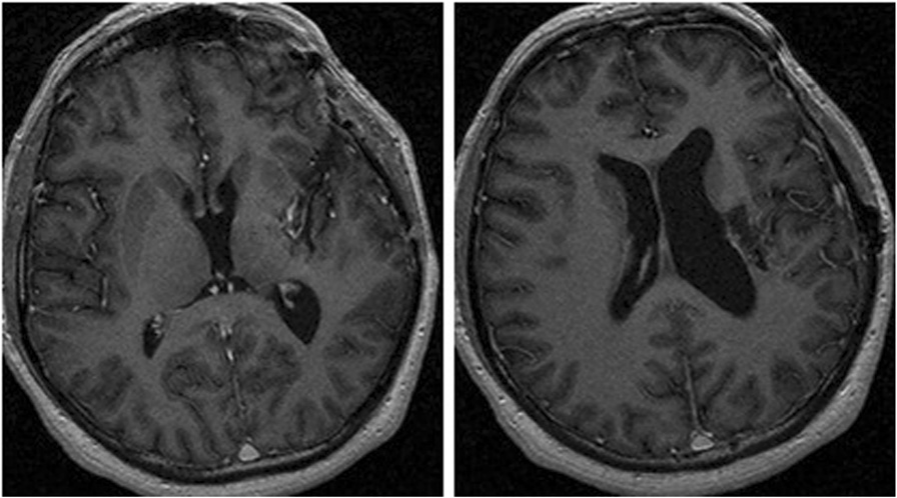
Control MRI (T1-enhancement) after 17 months of surgery. Post-surgical scar is presented in left insular region. No new intracerebral lesion

Due to histological result and extension study, the patient started the therapy with Dabrafenib 150 mg/12 h with good clinical and radiological result. No toxic effects appeared. After 5 months, Trametinib 2 mg/24 h was added to the treatment. After 18 months, the patient is stabilized and has no any new lesion nor therapy-related complication (Figs. 5 and 6). He is doing rehabilitation since the intervention and is independently walking.

### Discussion

Primary cerebral melanoma is an uncommon disease. CNS melanoma represents only 1 % of all melanomas and 0.07 % of all brain tumors [3]. Less than 30 cases have been reported in the literature between 1989 and 2016 [10, 11]. Leptomeningeal melanocytes come from neural crest, a group of multipotential cells that, on day 22 of embryogenesis, become leptomeningeal cells, glial cells, adrenal medulla cells, and melanocytes. The main location of melanocytes is in the piamatter around inferior surface of the cerebellum, spinal medulla, and high cervical medulla. According to Quillo-Olvera et al., the most common locations of primary brain melanoma are the lobe (53.1 %), posterior fossa (17.3 %), and pineal region (13.6 %) [12]. Vijayalaxmi et al. did a search between 1989 and 2014, and they found 19 cases of primary CNS melanoma, none of them at the insular region [11]. In our literature review made until 2016, we found at least four cases more of primary melanoma and none of these at the insular region. In contrast to brain metastasis, which usually occur in the junction between gray and white matter, intraparenchymal nodular lesion of primary melanomas may occur in the brain in any leptomeningeal location [3], as occurs in insular region close to Sylvian fissure. There are some cases in the literature, as reported by Pan et al., Greco Castro et al., and Rajes et al., about primary nodular lesion in the temporal lobe adjacent to the Sylvian fissure [2, 3, 10].

The common radiological characteristics of the cerebral melanomas (metastatics or primaries) in a MRI are hyperintense signal in T1 and hypointense in T2 due to the melanin paramagnetic effects and homogeneous enhancement on post-contrast images [13, 14]. On the contrary, in the presence of intralesional hemorrhage, all the sequences show a heterogeneous enhancement as it occurs in our case. In these situations, the radiological diagnosis is very complicated and the histology exam has to be considered. Shinomiya et al. explained the reason for these hemorrhages: intracranial melanomas have fenestrated endothelial cells promoting intralesional bleedings [15].

The differential diagnosis between primary melanoma and brain metastasis of melanoma is a controversial topic. The same radiological and histological findings make the real diagnosis extremely difficult [12]. Clinically, they presented equal. One difference between them, according to Terao et al., is the age. Primary CNS melanoma appears in relatively younger patients (usually under 50 years old) [16], as in our case. In 1976, Hayward proposed this classification: (1) primary brain malignant melanoma, (2) secondary brain malignant melanoma, and (3) other brain tumors with melanin [17]. He described the primary CNS melanoma when there are (1) no malignant melanoma outside CNS, (2) absences of this lesion in other part of CNS, and (3) histological confirmations of melanoma [8]. Since then, no other criteria have been developed [18] probably because the low incidences of primary CNS melanoma. In presented case, the patient had these three points and he was diagnosed of primary cerebral melanoma in unusual location after these points were checked.

It is well known that malignant melanomas have aggressive behavior and tend to metastasize to remote organs including lung, brain, bone, and lymph nodes. Braeuer et al. described some reasons for this: melanoma cells share numerous cell surface molecules with vascular cells, are highly angiogenic, are mesenchymal in nature, and possess a higher degree of “stemness” than other solid tumors do [7]. However, as Do-Hyoung et al. say, extracranial metastasis from primary CNS melanoma to lung, spleen, pancreas, and kidney has rarely been reported [8]. The literature has been reviewed and any relevant paper about this item has been found. In the case presented here, all the complementary tests made during hospitalization came negative (extension CT scan and skin-mucous analysis). Because of the rarity of primary brain melanoma, new tests were performed after discharge. The physical exam did not reveal any melanoma in the skin or mucous. FDG PET has shown to be sensitive for assessing metastases in lymph node and for detecting occult distant metastases in patient with malignant melanoma [9, 19, 20]. Although biopsy could be considered the gold standard for diagnosis, many papers have demonstrated the usefulness of 18F-FDG PET/CT in detecting lymph node metastasis. The volume of lymph node plays an important role in the accuracy and reliability of this test [9, 21]. Flavio et al. reported a sensitivity of 95 %, specificity, 84 %, positive predictive value, 92 %, negative predictive value, 89 %, and accuracy, 91 % [9]. Blessing et al. had a sensitivity (Se) 74 % and specificity (Sp) 93 % [22]. Aukema et al. have the best results in specificity (98 %), positive predictive value (PPV) (96 %), and accuracy (93 %). The Se was 87 % and negative predictive value (NPV) 91 % [23]. Jouvet et al. reported a Se, 79.8 %; Sp, 93.1 %; PPV, 93.2 %; and NPV, 79.4 % [24]. When the lymph node metastasis is >10 mm, Flavio et al. had a sensitivity of 100 %. Wagner et al. found a sensitivity of 90 % when the volume is >80 mm^3^ [21]. In the presented case, the lymph node metastasis had a 4.2 cm × 3.8 cm × 3.5 cm dimension (Fig. 4). All these results encourage the use of FDG PET in melanoma patients with possible lymph node involvement. Lumbar puncture is another complementary test in patients with CNS melanoma. Nevertheless, our patient did not have any suspicion of leptomeningeal spread in clinical presentation nor MRI test. The focal neurological deficit was secondary to the affected area. The MRI did not show hyperintense signal, punctuate, and/or linear enhancement in the sulci and gyri of the supra-infratentorial part of the brain [3, 8]. Due to the low probability of leptomeningeal affectation, the LP was not done.

Malignant melanoma has very poor prognosis because of its aggressive behavior. Nevertheless, the prognosis of primary CNS melanoma appear to be better than metastatic brain examples, particularly if localized and if complete resection is possible. In these cases, the median survival is 20.7 months [12]. In our case, despite we did not know the nature of the lesion before surgery, we made a complete resection of the lesion as it is shown in control image test (Fig. 6). In the literature review about treatment option made by Rajesh et al., they concluded that there are no any specific guidelines nor standard protocols. According to them, surgical excision is the mainstay of the treatment to better vital and functional prognosis. After surgery, adjuvant therapy with chemotherapy and, sometimes, radiotherapy play an important role, although these tumors are considered radio-resistant [10]. Approximately 45 % of melanomas have BRAF mutation [25]. In 2013, FDA approved Dabrafenib as a new option of treatment for metastatic melanoma. Dabrafenib, a BRAF inhibitor, target the MAPK pathway and improves, considerably, the prognosis of these patients with responses rates of 50 % and progression-free survival for 6 months [26]. Medina et al. reported that the combination of Dabrafenib and Trametinib results in a median overall survival of more than 2 years [27]. Long et al., in their study about the association between Dabrafenib + Trametinib, had 20 % patients progression free at 3 years [28]. According to literature results, our patient is stable after 18 months, independent for all the activities, and without new metastasis.

## Conclusions

Although malignant melanomas have poor prognosis, total surgical resection and new therapies are increasing the overall survival and improving the quality of life. According to our case report and literature review, in a patient with suspected primary brain melanoma, in spite of having extracerebral metastasis, aggressive treatment may be considered.

## Abbreviations

CNS, central nervous system; Fig, figure; LP, lumbar puncture; NPV, negative predictive value; PPV, positive predictive value; Se, sensitivity; Sp, specificity
